# Role of Hydrogen Sulfide, Substance P and Adhesion Molecules in Acute Pancreatitis

**DOI:** 10.3390/ijms222212136

**Published:** 2021-11-09

**Authors:** Ayush Kumar, Madhav Bhatia

**Affiliations:** Department of Pathology and Biomedical Science, University of Otago, Christchurch 8140, New Zealand; ayush.kumar@postgrad.otago.ac.nz

**Keywords:** ICAM-1, VCAM-1, MAdCAM-1, VAP-1, Hyaluronan, hydrogen sulfide, substance P, acute pancreatitis, selectins

## Abstract

Inflammation is a natural response to tissue injury. Uncontrolled inflammatory response leads to inflammatory disease. Acute pancreatitis is one of the main reasons for hospitalization amongst gastrointestinal disorders worldwide. It has been demonstrated that endogenous hydrogen sulfide (H_2_S), a gasotransmitter and substance P, a neuropeptide, are involved in the inflammatory process in acute pancreatitis. Cell adhesion molecules (CAM) are key players in inflammatory disease. Immunoglobulin (Ig) gene superfamily, selectins, and integrins are involved at different steps of leukocyte migration from blood to the site of injury. When the endothelial cells get activated, the CAMs are upregulated which leads to them interacting with leukocytes. This review summarizes our current understanding of the roles H_2_S, substance P and adhesion molecules play in acute pancreatitis.

## 1. Introduction

The main objective behind this review is that we highlight a key role of hydrogen sulfide, substance P, and cell adhesion molecules in inflammation of pancreas.

To the best of our knowledge, this is the first review to highlight these the roles of specific cell adhesion molecules from Immunoglobulin superfamily cell adhesion molecule (IgSF CAM), as well as a couple of atypical adhesion molecules (Hyaluronan and Vascular Adhesion Protein-1/Amine Oxidase Copper Containing 3 (VAP-1/AOC3)) in acute pancreatitis and associated inflammatory response.

Acute pancreatitis is an inflammatory disorder of the pancreas that is associated with significant morbidity and mortality. It is characterized by acute inflammation; the severity can vary between mild acute pancreatitis with local edema to severe disease with widespread parenchymal necrosis with severe hemorrhage. Mild acute pancreatitis can be reversible. However, in cases of high severity, acute pancreatitis is associated with systemic inflammatory response syndrome (SIRS), which, if excessive, can lead to multiple organ dysfunction syndrome (MODS) (a major component of which is lung injury, clinically manifested as acute respiratory distress syndrome—ARDS) and even death [1].

Hydrogen sulfide (H_2_S) and substance P are key mediators of inflammation in acute pancreatitis. Recruitment of leukocytes to the site of injury/inflammation by adhesion molecules is a key step in the regulation of the inflammatory process. There is evidence for the vital role played by adhesion molecules in the pathogenesis of inflammation in acute pancreatitis. This review describes our current understanding on the role of H_2_S and substance P and their interaction with adhesion molecules in the regulation of inflammation in acute pancreatitis

## 2. Acute Pancreatitis

The prevalent etiological factors leading to the development of acute pancreatitis are pancreatic ductal obstruction due to gallstones and excessive alcohol consumption, which together are responsible for over half (50%) of the reported cases. A medical procedure called endoscopic retrograde cholangiopancreatography (ERCP) and common drugs such as thiazide diuretics, can also trigger pathological cellular pathways and organelle dysfunction, leading to characteristic pathology of acute pancreatitis, which involves acinar cell death accompanied by local and systemic inflammation.

In the industrialized world, acute pancreatitis affects 10–20 per 100,000 people. On the global scale, acute pancreatitis affects 34 people per 100,000, and the incidence is increasing [1,2]. Currently, it is the most common gastrointestinal disorders to cause hospitalization in the USA, generating an annual bill of $9.3 billion for the healthcare system [3,4]. Around 18% of the people suffering from acute pancreatitis experience recurrence, and 8% of people go on to develop chronic pancreatitis. Both of these together add to the financial burden on healthcare [5,6]. In 2013, readmissions costs resulting from acute pancreatitis exceeded $3.8 billion in the USA [7].

## 3. Inflammatory Mediators

Acute pancreatitis, as the name suggests, is an inflammatory disease with a rapid onset. Like other inflammatory conditions, different mediators, such as hydrogen sulfide, substance P and adhesion molecules, play critical roles at various stages of disease progression.

### 3.1. Hydrogen Sulfide (H_2_S)

Hydrogen sulfide (H_2_S) is a colorless, poisonous, and toxic gas with a foul odor. It is a biological gaseous signaling molecule, which plays an immense pathophysiological role in various diseases [8]. Endogenously synthesized H_2_S has been implicated in various physiological and pathological aspects of an inflammatory response [9]. H_2_S is also implicated in increasing the generation of different pro-inflammatory mediators and worsening systemic inflammation.

#### 3.1.1. Synthesis

There are 2 main sources through which H_2_S is synthesized:

**Enzymatic source**

There are currently three known enzymes that are capable of synthesizing hydrogen sulfide: cystathionine-γ-lyase (CSE), cystathionine-β-synthase (CBS), and 3-mercaptopyruvate sulfurtransferase (3MST). Both CBS and CSE are found in the cytosol, and 3MST is found in the mitochondria [10]. The tissue distributions of these three enzymes are varied in location as well as levels of expression. The liver and kidney are the two organs that express all three enzymes at high levels [11,12,13]. CBS is also expressed in the brain, lung, stomach, and pancreas [11] while CSE is found in the stomach, small intestine, pancreas, and smooth muscle [9,13,14]. 3-MST in shown to be present in the brain, heart, lung, thymus, and testes [12]. 

The levels of CSE and CBS in tissues where both enzymes are co-expressed are varied. For example, the liver and kidney express higher levels of CSE compared to CBS, 60 and 20-fold respectively [15]. 

**Figure 1:**
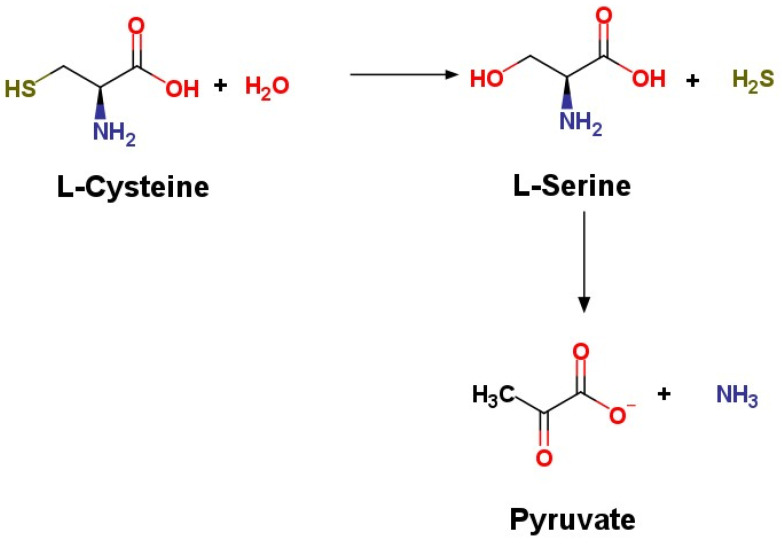
H_2_S synthesis via CSE.

CSE and CBS are enzymes involved in the transsulfuration pathway and are directly involved in the generation of hydrogen sulfide as a byproduct through their diverse catalyzed reactions [16]. Under physiological conditions, the major reaction catalyzed by CSE in the generation of hydrogen sulfide is via the α,β-elimination of cysteine [17], which is a sulfur containing (as a thiol group) amino acid (Figure 1).

**Figure 2:**
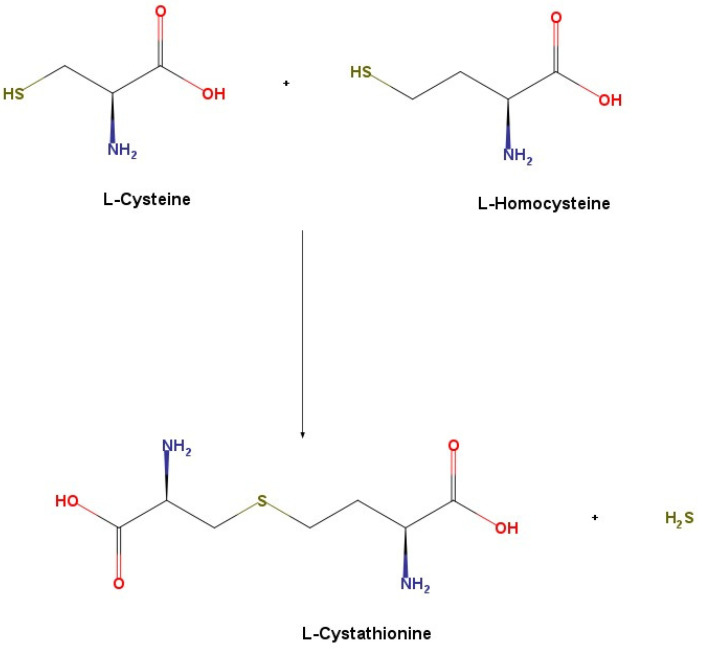
H_2_S synthesis via CBS.

The major reaction catalyzed by CBS in the generation of hydrogen sulfide is via the condensation of cysteine and homocysteine to produce cystathionine [16] (Figure 2).

3MST, on the other hand, catalyzes the conversion of 3-mecaptopyruvate to pyruvate resulting in the formation of a 3MST persulfide. This persulfide moiety then requires specific reducing agents such as thioredoxin and dihydrolipoic acid to release hydrogen sulfide [18]. Therefore, it is perceived that the major contributors of endogenous hydrogen sulfide are CSE and CBS [15] although it has been shown that 3MST does contribute to endogenous hydrogen sulfide generation in certain tissues [19,20].

**Labile source**

Apart from enzymatic synthesis of free hydrogen sulfide, there are bound sulfur sources that could release free hydrogen sulfide upon acidification or reduction of the parent molecule; these sources are acid-labile sulfide and Dithiothreitol (DTT)-labile sulfide (or sulfane sulfur) respectively [21]. The relative amounts of free sulfide present in these discreet pools in biological samples are still a subject of continuous study as more sensitive and specific methods are continuously developed. At present, human plasma acid-labile sulfide is reported to be in the low micromolar range followed by sulfane sulfur and free sulfide, both of which are at similar low nanomolar concentrations [22]. In murine tissues, the brain was reported to contain 12.5 nmol/g and 18.5 nmol/g of acid labile and sulfane sulfur respectively [23]. 

The major source of acid-labile sulfide is iron-sulfur cluster containing proteins [24], these proteins are ubiquitous, diverse and serve a wide array of functions [25]. Although the iron-sulfur cluster containing proteins provides a substantial source of labile sulfide, it is an unlikely physiological endogenous source as a maximum of pH 5.4 is required to release the bound sulfur as sulfide [26]. Sources of sulfane sulfur include thiosulfate, thiosulfonates, polysulfides, and protein persulfides [24]. Although reducing agents are present in the cell (i.e., glutathione and cysteine), it was demonstrated that an alkalized cytoplasm is instrumental for an efficient release of hydrogen sulfide from these sulfane sulfur sources [26]. This was shown by using rat astrocytes, which have high concentrations of extracellular K^+^ in physiological conditions when adjacent or nearby neurons are in excited state [26]. Among the available sources of sulfane sulfur, protein persulfides are particularly intriguing as they are not synthesized de novo as a persulfide but are a product of a cystiene thiol modification by the addition of divalent sulfide molecule resulting in the formation of an R-S-SH; this process is termed sulfhydration akin to s-nitrosylation [27]. A substantial number of proteins were reported to be basally s-sulfhydrated and the addition of exogenous hydrogen sulfide further increases the level [27]. 

H_2_S has also been shown to be scavenged in proteinaceous biological solutions such as plasma [28], tissue homogenates [26,29] and single protein solutions containing cysteine residues [28]. These scavenged hydrogen sulfides were then partially retrieved by applying a reducing agent to the homogenates [26,29]. This suggests that protein persulfides could serve as both a source as well as a sink of endogenous free hydrogen sulfide.

**Figure 3:**
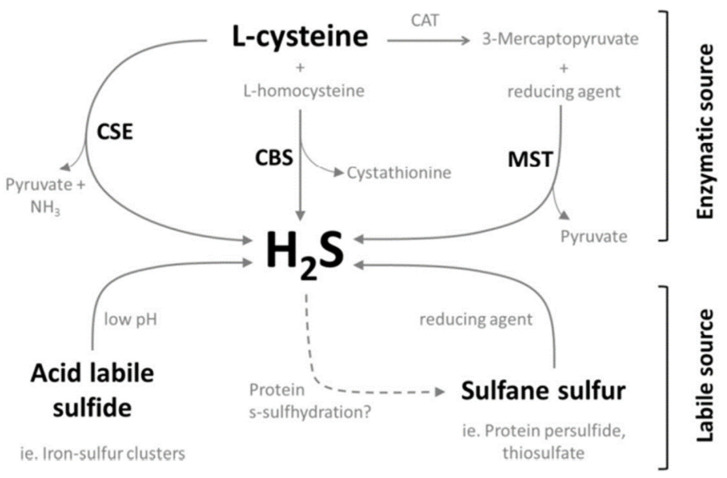
Layout of H_2_S synthesis via enzymatic and labile sources.

Figure 3 provides an overview of our current understanding regarding the endogenous synthesis of H_2_S.

#### 3.1.2. Role in Acute Pancreatitis

Exploring the mechanism through which H_2_S affects the inflammatory process in acute pancreatitis has been of significant scientific interest. This pursuit involves working with both isolated pancreatic acini, which is an ideal in vitro model for studying the exocrine function of pancreas, and also an in vivo model of acute pancreatitis. 

Stimulation of acini by caerulein in vitro leads to changes within them, similar to those observed in acini in acute pancreatitis in vivo. In isolated pancreatic acinar cells, inhibition of CSE by propargylglycine (PAG), decreases production (messenger RNA (mRNA) as well as protein) of the chemokines monocyte chemoattractant protein (MCP)-1, macrophage inflammatory protein (MIP)-1α, and MIP-2 [30,31]. 

A pro-inflammatory action of H_2_S in acute pancreatitis has been shown using different and complementary approaches: pharmacological inhibition of H_2_S synthesis by CSE [32], gene deletion using CSE knockout mice [33], and CSE gene silencing using siRNA [34]. It was shown that when CSE is inhibited by PAG resulting in decreased H_2_S production in caerulein-induced acute pancreatitis in the mouse in vivo, inflammation is inhibited through downregulation of MCP-1, MIP-1α, and MIP-2 expression [31]. Another study has shown that the proinflammatory actions of H_2_S in acute pancreatitis are mediated via PI3K/Akt/Sp1 signaling pathway [35]. Recent reports have shown that the attenuation of CSE mediated H_2_S synthesis with gene silencing, gene deletion, and pharmacological inhibition alleviates inflammation [9]. 

A recent study has suggested that autophagy, a key pathology in acute pancreatitis, is enhanced by hydrogen sulfide by the AMPK/mTOR pathway [36]. 

### 3.2. Substance P (SP)

Substance P is a neuropeptide from the tachykinin family. This family comprises of 4 other members, namely neuropeptide K (NPK), neuropeptide-*γ* (NP*γ*), neurokinin-A (NKA), and neurokinin-B (NKB). Substance P is an important mediator for inflammation in acute pancreatitis and associated lung injury. It is synthesized in cell bodies of vagal sensory ganglia and transported bidirectionally toward the CNS and thoracic and abdominal viscera. It is also the most abundantly available neuropeptide [37,38]. Substance P acts via three different G protein coupled receptors (GPCRs), namely neurokinin (NK) 1R, 2R, and 3R (R = receptor). Of these three, substance P has highest affinity towards NK-1R, while having a minimal affinity for NK-2R and NK-3R. Hence, the NK-1R is responsible for majority of the effects brought on by substance P [39].

Substance P is present along with other amine neurotransmitters and/or peptides in neuronal terminals. When released, it acts as a neurotransmitter or a neuromodulator. In the peripheral nervous system, substance P acts as a neurotransmitter in the primary sensory neurons with cell bodies in the dorsal root ganglia and cranial sensory ganglia. These neurons are responsible for transmitting sensory information from the periphery to the central nervous system, along with the local release of substance P. This leads to neurogenic inflammation, which is characterized by vasodilation and increased vascular permeability [37,38].

#### Role in Acute Pancreatitis

Substance P, an important inflammatory mediator, is critical in the pathogenesis of acute pancreatitis. To demonstrate this, studies were done which showed its role acting via the NK-1R [40,41,42,43]. 

Different experimental approaches—gene knockout for substance P (*pre-protachykinin-A—PPT-A* gene) and its receptor [41] NK-1R, pharmacological inhibition of its action by using specific receptor antagonist [44], and inhibitors for neutral endopeptidase, the enzyme responsible for its inactivation [45] have been used to study the role of substance P in acute pancreatitis. 

Substance P induces local vasodilatation, increases microvascular permeability and edema, which lead to the accumulation of leukocytes. Substance P is also produced by macrophages, eosinophils, and dendritic cells. Using isolated pancreatic acini and in vivo models of acute pancreatitis, our group has shown that Substance P stimulates the formation of pro-inflammatory chemokines by a Ca^2+^, protein kinase C (PKC-δ), extracellular-signal-regulated kinase (ERK), S locus receptor kinase (SRK), and nuclear factor kappa B (NF-κB) dependent pathways [46,47].

### 3.3. Interaction between H_2_S and Substance P

Hydrogen sulfide and substance P interact with each other and regulate the development and progression of acute pancreatitis. However, more can be investigated about their relationship and acute pancreatitis. One such study showed that inhibition of substance P by 2 different methods; NK-1R antagonism using CP-96,345 and PPT-A^−/−^ mice, which lack the gene responsible for substance P synthesis, led to a decrease in hydrogen sulfide mediated lung inflammation [48], suggesting a relation between them. 

When normal mice were injected with sodium hydrogen sulfide (NaHS) intraperitoneally, a significant NK-1R-dependent increase in plasma levels of substance P was observed, while pronouncing lung inflammation and acute pancreatitis [8]. In *preprotachykinin A (PPT-A*—substance P encoding gene) knockout (*PPT-A^−/−^*) mice, this inflammatory effect of H_2_S on lung inflammation was not observed. 

Removal of substance P from sensory neuron by the administration of capsaicin, protected mice against H_2_S-induced lung inflammation. Furthermore, administration of capsazepine, an antagonist of the transient receptor potential vanilloid-1 (TRPV-1), protected mice against H_2_S-induced inflammation. These findings suggest that substance P plays a critical role in neurogenic inflammatory pathways involved in H_2_S-mediated inflammation [48]. Substance P is present in a number of pathways within the central and peripheral nervous system and also in various cells of the immune system [49].

As acute pancreatitis progresses, there is an increased risk for the development of SIRS, which is similar to the infection-dependent SIRS caused in sepsis. A significant decrease of substance P in plasma and tissue, along with decreased NK-1R expression following SIRS caused by sepsis in CSE- knockout mice is observed, which strongly suggests that CSE/H_2_S is one of the key mechanisms regulating substance P and NK-1R in sepsis. This is consistent with studies using CSE inhibitors and H_2_S donors [9]. 

Another study to explore H_2_S-substance P relationship was carried out by inhibiting H_2_S synthesis with the help of PAG. The treatment resulted in decreased levels of substance P in the pancreas, lung, and plasma in acute pancreatitis [50]. This pharmacological inhibition of H_2_S also reduced expression for both NK-1R and *PPT-A* in the pancreas and lungs in acute pancreatitis [50]. These findings suggest that the proinflammatory actions of H_2_S in acute pancreatitis may be mediated via substance P [50]. 

In vitro studies with isolated pancreatic acini indicate that in acute pancreatitis, H_2_S enhances the activity of the Toll-like receptor 4 pathway and NF-κB via substance P [51].

### 3.4. Role of Endothelial Cells in Acute Pancreatitis

Under physiological conditions, vascular endothelial cells play a crucial role in regulating vascular wall functions. However, in the case of acute pancreatitis, there is disturbance in microcirculation, leading to endothelial cell injury. There are different factors released in such cases, for e.g., thrombomodulin, vasodilators, vasoconstrictors, and adhesion molecules.

In acute pancreatitis, different chemical mediators are produced in excess, which leads to the accumulation of leukocytes at the site of injury (predominantly neutrophils) and dysfunction in various organs [52]. Neutrophils and pancreatic parenchymal cells are acted upon by endotoxins and cytokines, which upregulates the expression of adhesion molecules and reinforces their adhesion potential. Chemokines, such as Interleukin-8 (IL-8), increase the adhesion potential of immune cells on the vascular endothelial cells, which are immobilized on the cell surface and migrate through the spaces to the inflamed area [53,54].

### 3.5. Adhesion Molecules

Adhesion molecules like selectin, integrins, and immunoglobulins have a pivotal role in the inflammatory process. Adhesion molecules are instrumental in cell migration, cell proliferation, signal transduction, as well as in the development and repair at the tissue level. They are important in mediating the infiltration of leukocytes from the bloodstream to the inflammatory site and serve to enable an orderly sequence of cell–cell interactions that sustain leukocyte adherence to vascular endothelium and the subsequent trans endothelial migration into inflamed tissue, for e.g., in acute pancreatitis [55].

In acute pancreatitis, inflammation is characterized by migration of inflammatory mediators and structural disruption of tissue. The disease progression involves an increase in solute permeability, followed by the development of interstitial edema. This change in permeability results from a decrease in intercellular adhesion among pancreatic acini and/or endothelial cells [55]. 

One of the key regulators in acute pancreatitis is oxidative stress. It promotes the expression of adhesion molecules in the inflamed area. The expression of these cell adhesion molecules is also upregulated following endothelial cell activation by different inflammatory chemokines and cytokines. When endothelial cells are activated, their interaction with leukocytes is increased. Selectins are important mediators for the initial interaction between the leukocytes and active endothelial cells are selectins; while other adhesion molecules, such as integrins and Ig superfamily cell adhesion molecules (IgSF CAM) are important for the firm cellular adhesion and following steps [55]. 

Different adhesion molecules, such as vascular cell adhesion protein 1 (VCAM-1), intercellular adhesion molecules 1 (ICAM-1), vascular adhesion protein1 (VAP-1), Hyaluronan, and mucosal addressin in cell adhesion molecules (MAdCAM-1) have been reported in the inflammation of the pancreas, resulting in the progression of inflammatory disease [56,57,58,59,60]. 

The severity of acute pancreatitis can be reduced by inactivation and/or immunoneutralization of adhesion molecules [61,62] It has been observed in rodents that following inactivation of adhesion molecules by monoclonal antibodies, capillary blood flow is increased in the pancreas, along with reduction in leukocyte rolling, and stabilization of capillary permeability [63]. The interaction between leukocytes and endothelial cells through adhesion molecules is a part of early events in acute pancreatitis, where it determines the rate for microvascular dysfunction. The therapeutic potential of inhibiting adhesion molecules is in the initial phases of investigation. However, new chemical agents that target these inflammatory mediators may soon get tested in a clinical setting.

Table 1 summarizes our knowledge on the biological effects of adhesion molecules in acute pancreatitis.

#### 3.5.1. ICAM-1 

High serum levels of ICAM-1 have been detected in acute pancreatitis, especially in severe and/or necrotizing acute pancreatitis. These elevated ICAM-1 levels can be correlated with a higher mortality rate and the development of pancreatic necrosis. Hence, it is a potential early marker, for both the diagnosis as well as prognosis of severe acute pancreatitis [64].

Neutrophils are consequential for inflammation in acute pancreatitis. To explore their role, mice were treated with an anti-neutrophil serum. Following administration, the treated mice were protected against acute-pancreatitis-associated lung injury [41], demonstrating their importance.

A vital role of ICAM-1 in the action of neutrophils in acute pancreatitis was shown in a study in which genetically deficient mice in ICAM-1 were protected against acute pancreatitis and associated lung injury. In ICAM-1 knockout mice, treatment with an anti-neutrophil serum did not provide any further protection [65]. These results showed an important contribution of ICAM-1 in the action of neutrophils in inflammation in acute pancreatitis. ICAM-1 and chemokines such as macrophage inflammatory peptide 1-α (MIP 1-α), MIP-2, and interleukin-8 (IL-8) are responsible for the recruitment of neutrophils, both in the pancreas and the lungs. These events are considered vital in the initial development of acute pancreatitis [66,67].

Treatment with a rapid H_2_S donor, such as sodium hydrogen sulfide (NaHS), results in increased expression of ICAM-1 and neutrophil adhesion to pancreatic acinar cells treated with caerulein, via NF-κB and Src-family kinase pathway [68]. 

#### 3.5.2. VCAM-1

VCAM-1 is usually expressed when endothelial cells are activated, and not in normal conditions [55]. The expression of VCAM-1 is upregulated under stimulation from different inflammatory cytokines. It plays an important role in the migration of various leukocytes, namely monocytes, eosinophils, basophils, lymphocytes, and natural killer (NK) cells, to the inflamed area [69]. Blockage of VCAM-1 resulted in decreased recruitment and adherence of leukocytes into the lungs, thus inhibiting lung injury in severe acute pancreatitis [70]. VCAM-1 expression is known to be positively correlated with the extent of organ damage in the animal model of acute pancreatitis [71].

#### 3.5.3. MAdCAM-1

MAdCAM-1 is present on high endothelial venules and mucosal vessels, where it is responsible for directing lymphocytes toward Peyer’s patches and the intestine [72]. MAdCAM-1 executes critical roles in different inflammatory diseases, e.g., in inflammatory bowel disease (IBD). MAdCAM-1 has the capacity to bind and retain β7-integrin expressing lymphocytes within the gut, where they appear to exacerbate inflammation in IBD. 

The expression of MAdCAM-1 on pancreatic vascular endothelium has been recorded in caerulein-induced acute pancreatitis. This suggests that MAdCAM-1 might play a role in the recruitment of lymphocytes, resulting in the exacerbation of both local injury and remote injury to multiple organs in acute pancreatitis [60]. Different reports suggest that the expression of MAdCAM-1 in pancreas is regulated via protein kinase C (PKC), mitogen-activated protein kinase (MAPK), NF-kB, etc., along the lines of other adhesion molecules in colitis and IBD [54,73].

#### 3.5.4. VAP-1

VAP-1, also known as amine oxidase, copper containing 3 (AOC3), is an endothelial molecule which has both adhesive and enzymatic properties in vitro. Studies have shown that VAP-1 is instrumental in leukocyte migration, observed by inhibiting VAP-1 in vivo. The inhibition of VAP-1 inhibited the migration of lymphocytes, granulocytes, or macrophages into inflamed pancreas [56]. Some reports characterize the role of VAP-1 in regulating granulocyte adhesion to tissue vasculature and lymphocyte binding to High Endothelial Venules (HEVs) at the site of inflammation, for instance in reperfusion injury from myocardial infarction. This was observed by inhibiting human VAP-1 by monoclonal antibodies in vitro [74], indicating the role of VAP-1 in inflammatory diseases. There is considerable in vitro evidence implicating VAP-1 for leukocyte trafficking in humans [75]. 

#### 3.5.5. Hyaluronic Acid or Hyaluronan 

Deposition of extracellular matrix components has been observed in both acute and chronic pancreatitis. Multiple reports have shown that hyaluronic acid gets accumulated in the local tissue during different inflammatory conditions, such as in alveolitis, myocarditis, and Crohn’s disease [76,77,78]. These findings indicate that the alteration of hyaluronan content in acute pancreatitis is a possibility as well. Hyaluronan is highly hydrophilic in nature, which is demonstrated by its potent water-binding capacity. Accumulation of hyaluronan in tissue and the development of interstitial edema have been positively correlated in several experimental models. The resulting edema can result in increased interstitial pressure, disturbed microcirculation, and finally tissue necrosis in pancreatitis [58,79]. 

#### 3.5.6. Selectins

The selectin family comprises of three members, namely endothelial selectin (E-selectin), leukocyte selectin (L-selectin), and platelet selectin (P-selectin).

During acute pancreatitis, the damaged pancreatic tissue releases xanthine oxidase. It is responsible for the generation of free radicals, leading to increased oxidative stress. This is responsible for the upregulation of P-selectin in the pulmonary endothelium. High levels of P-selectin are positively correlated with leukocyte recruitment at the site of tissue injury [62]. Inhibition of xanthine oxidase can potentially inhibit the upregulation of P-selectin expression, the infiltration of neutrophils, and ameliorate the progression of pancreatitis-associated lung injury (PALI) in rats [62]. 

It was found that the plasma and tissue levels of E-selectin and P-selectin are elevated in severe acute pancreatitis. This finding can be instrumental in determining the progression of acute pancreatitis [80,81]. Increased expression of E- and/or P-selectin is positively correlated with an increased risk of death, longer period of hospitalization, and development of tissue necrosis in pancreas [81,82]. There is a high risk of respiratory failure following severe acute pancreatitis, caused by PALI. Its progression is regulated by different selectins, especially P-selectin [83,84,85]. 

Neutrophil recruitment and extravasation at sites of inflammation are partly dependent on the endothelial expression of P-selectin and ICAM-1. It was observed in various experimental conditions that treatment with antibodies targeting these adhesion molecules conferred a protective effect [62]. Treatment with an anti-P-selectin antibody inhibited neutrophil infiltration into the pancreatic parenchyma, and also suppressed tissue inflammation as well as tissue necrosis [86,87].

A study has established the link between substance P and selectins in acute pancreatitis, by demonstrating that the administration of CP-96,345 (a NK-1R antagonist) significantly reduced mRNA and protein expression of E-selectin and P-selectin [88].

#### 3.5.7. Other Adhesion Molecules

There are other adhesion molecules that have been suggested to a role at various stages in acute pancreatitis. They are at tight junction (occludin [89], claudins [89,90,91,92]), junctional adhesion molecules (JAM-A, -B, and -C) [92,93,94], zonula occludin (ZO-1, -2, and -3) [89] and adherens junctions (catenins, cadherins) [95,96].

Tricellulin is an adhesion molecule present in the pancreas, whose activity in acute pancreatitis has not yet been shown. It is present at tight junctions [97].

## 4. Conclusions

**Figure 4:**
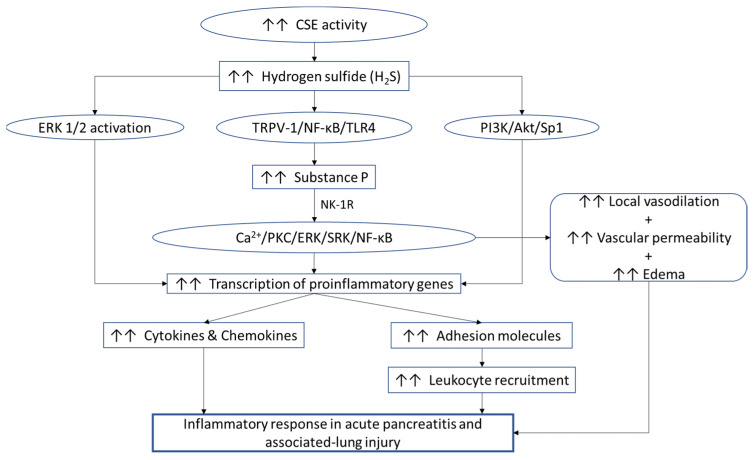
Interaction of H_2_S, substance P, and adhesion molecules in the regulation of inflammatory response in acute pancreatitis.

In this review we have discussed how H_2_S, substance P and adhesion molecules play an important role in acute pancreatitis. Figure 4 summarizes our current understanding of the contribution of the subject. Different in vivo animal models of human diseases and various in vitro models have significantly contributed to our understanding of the roles H_2_S, substance P and adhesion molecules play in the process of inflammation in acute pancreatitis. However, the various studies on these mediators have also left current and prospective future researchers on the topic to explore different things that are still unknown.

Despite advances on the molecular mechanisms of H_2_S, substance P and adhesion molecules, there is a lot left to be understood on how these mediators work as well as interact with each other in acute pancreatitis. If these mediators can be effectively targeted pharmacologically, these would be game changers in the treatment of acute pancreatitis, which is currently treated by providing palliative care and does not have many viable and patient friendly treatment options. Work on them will also extend into different inflammatory diseases and translate to a better understanding of different types of inflammatory diseases. Hence, more work can help facilitate the translation of this knowledge from the bench to the bedside, which is of critical importance.

## Figures and Tables

**Table 1:** Biological effects of adhesion molecules in acute pancreatitis.

Adhesion Molecule	Biological Effects
ICAM-1 [41,64,65,66,67,68]	Important marker for early detection, key role in neutrophil migration
VCAM-1 [55,69,70,71]	Important role in leukocytic migration
MAdCAM-1 [54,60,72,73]	Possible role in lymphocytic migration
VAP-1 [56,74,75]	Important role in leukocytic migration
Hyaluronan or Hyaluronic acid [58,76,77,78,79]	Key role in interstitial edema, which leads to tissue necrosis
Selectins [62,80,81,82,83,84,85,86,87,88]	Important for leukocyte recruitment

## Data Availability

Not applicable.
